# Gut microbial H_2_S promotes metabolic dysfunction in mice via hepatic PPARα suppression

**DOI:** 10.1093/ismejo/wrag226

**Published:** 2026-09-02

**Authors:** Shibo Lei, Xuan Qiu, Zhen Wang, Zuyang Zhang, Andong Zha, Yaying Zhou, Hao Chen, Jing Huang, Zheng Yu

**Affiliations:** Human Microbiome and Health Group, Department of Microbiology, Xiangya School of Basic Medical Sciences, Central South University, Changsha 410000, China; Human Microbiome and Health Group, Department of Parasitology, Xiangya School of Basic Medical Sciences, Central South University, Changsha 410000, China; Hebei Medical University Clinical Medicine Postdoctoral Research Station, Shijiazhuang 050000, Hebei, China; Department of Endocrinology, Third Hospital of Hebei Medical University, Shijiazhuang 050000, Hebei, China; Human Microbiome and Health Group, Department of Microbiology, Xiangya School of Basic Medical Sciences, Central South University, Changsha 410000, China; Human Microbiome and Health Group, Department of Microbiology, Xiangya School of Basic Medical Sciences, Central South University, Changsha 410000, China; Department of Spine Surgery, The Second Xiangya Hospital, Central South University, Changsha 410000, Hunan, China; Human Microbiome and Health Group, Department of Microbiology, Xiangya School of Basic Medical Sciences, Central South University, Changsha 410000, China; Human Microbiome and Health Group, Department of Parasitology, Xiangya School of Basic Medical Sciences, Central South University, Changsha 410000, China; Human Microbiome and Health Group, Department of Microbiology, Xiangya School of Basic Medical Sciences, Central South University, Changsha 410000, China; Human Microbiome and Health Group, Department of Parasitology, Xiangya School of Basic Medical Sciences, Central South University, Changsha 410000, China; Human Microbiome and Health Group, Department of Microbiology, Xiangya School of Basic Medical Sciences, Central South University, Changsha 410000, China

**Keywords:** hydrogen sulfide, metabolic disorders, gut microbiome, liver, PPAR

## Abstract

This study investigates the role of gut microbiota-derived hydrogen sulfide (H_2_S) in obesity and glucose metabolism disorders. By integrating human gut metagenomic data, intervention experiments in mouse models, and in vitro cellular assays, we identified a signature of microbial sulfur metabolism in human cohorts and provided experimental evidence for its causal role and underlying metabolic mechanisms in mice. In clinical cohorts with obesity and glucose metabolism disorders, we observed a notable enrichment of genes involved in sulfur transport and H_2_S production. In mouse models, administration of H_2_S-producing *Desulfovibrio desulfuricans*, engineered *Escherichia coli* expressing *phsABC*, and the H_2_S donor NaHS consistently induced body weight gain and impaired glucose tolerance. Transcriptome analysis and cellular experiments indicated that H_2_S was associated with downregulation of the peroxisome proliferator-activated receptor (PPAR) signaling pathway and lipid metabolism pathways in the liver, which may contribute to the abnormal accumulation of lipids and glycogen. Furthermore, rescue experiments using a PPAR agonist and an H_2_S adsorbent partially reversed these metabolic abnormalities. Collectively, our work provides experimental evidence in mouse models demonstrating that gut microbial H_2_S promotes metabolic dysfunction through hepatic PPARα suppression, providing potential targets for microbiome-based therapeutic interventions.

## Graphical abstract

**Graphical abstract:**
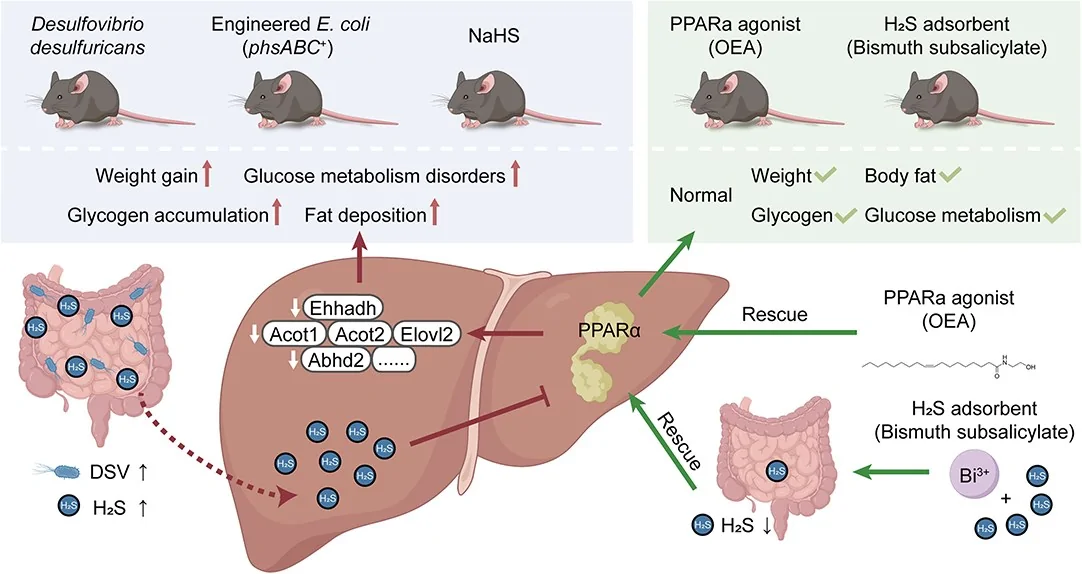

## Introduction

The global prevalence of obesity and type 2 diabetes mellitus (T2DM) has reached pandemic proportions, presenting a formidable public health challenge of the 21st century [1, 2]. These interconnected metabolic disorders are characterized by chronic hyperglycemia, insulin resistance, and dysfunctional lipid homeostasis, which collectively drive systemic complications such as cardiovascular disease and metabolic dysfunction-associated steatotic liver disease [3]. While excessive caloric intake and sedentary lifestyles remain primary drivers, accumulating evidence underscores the pivotal role of the gut microbiome as a dynamic interface between dietary inputs and host metabolic health [4]. Trillions of commensal microorganisms encode a metabolic repertoire that far exceeds the genomic capacity of the human host, generating a diverse array of bioactive metabolites—such as short-chain fatty acids, bile acids, and amino acid derivatives—that profoundly influence systemic physiology and energy balance [5].

Among these metabolites, hydrogen sulfide (H₂S) has emerged as a molecule of a dual nature [6]. Endogenously, H₂S is produced by host enzymes (eg, cystathionine-β-synthase， cystathionine-γ-lyase) and functions as a crucial gasotransmitter that modulates vasodilation, redox signaling, and inflammation [7, 8]. However, the gut lumen serves as a significant reservoir for H_2_S, primarily generated by specialized sulfate-reducing bacteria (SRB) through the reduction of dietary inorganic sulfate and sulfonated amino acids [9]. While low concentrations of H₂S are essential for maintaining the colonic mucus layer, its overproduction is increasingly associated with gut barrier disruption, mitochondrial inhibition, and localized mucosal inflammation [10, 11]. Despite this, a direct causal link between gut microbial H₂S production and the pathogenesis of systemic metabolic disorders remains poorly defined, representing a critical knowledge gap at the intersection of microbiology and endocrinology.

The human gut harbors specialized prokaryotes capable of reducing inorganic sulfate to H₂S, with members of the genus *Desulfovibrio* being the most prevalent and metabolically active SRB in the colon [12]. Their abundance is influenced by diet in healthy populations, particularly the intake of sulfur-containing compounds and fats [9, 13]. Clinically, however, alterations in the gut microbiome, including the enrichment of SRB [14] and sulfur metabolism [15], have been observed in cohorts with obesity and T2DM. Moreover, elevated H₂S levels have been reported to varying degrees in diabetic and obese individuals as well as in animal models of these conditions [15–18]. These associative studies, while suggestive, fall short of establishing causality. The central unresolved question is whether the observed microbial dysbiosis and elevated H₂S are merely secondary epiphenomena of an altered metabolic state or are active contributors to disease progression. Disentangling this requires moving beyond correlation to direct experimentation. Specifically, it remains unclear whether the metabolic phenotypes observed in the host are driven by the physical presence of the bacteria themselves, the specific enzymatic activity of their reductase complexes, or the terminal H_2_S metabolite. Addressing this ambiguity is essential for developing targeted therapeutic interventions that can mitigate the metabolic impact of a “sulfidogenic” microbiome.

In this study, we hypothesize that gut microbiota-derived H₂S is a causal effector in the development of obesity and glucose metabolism disorders, acting through the modulation of genes involved in lipid and carbohydrate metabolism. To rigorously test this hypothesis, we integrated human cohort bioinformatics with stepwise causal experiments in mouse models. First, we analyzed publicly available gut metagenomic data from 1580 individuals, identifying a conserved enrichment of microbial sulfur relay system genes in both obesity and glucose metabolism disorder cohorts. Guided by this human signature, we confirmed elevated fecal H₂S production in diet-induced obese mice. We then examined these interventions in separate experimental protocols using the prototypical SRB *Desulfovibrio desulfuricans*, engineered H_2_S-producing *Escherichia coli* expressing the thiosulfate reductase operon *phsABC* as a surrogate tool for local H_2_S generation, the H_2_S donor NaHS. This stepwise design provided convergent experimental evidence from distinct H_₂_S-related interventions. Coupled with in-depth phenotyping, hepatic RNA-seq analysis and cell experiment, this strategy provided convergent evidence and helped delineate the underlying hepatic response.

Our findings demonstrate that all three interventions sufficiently induced weight gain and glucose intolerance in mice, supporting a causal role for microbial H₂S. We further show that H₂S leads to abnormal accumulation of hepatic glycogen and fat. Transcriptomic analysis revealed that H₂S downregulates key genes in the hepatic PPARα signaling pathways, while concurrently disturbing the expression of multiple enzyme-encoding genes critical for lipid metabolism. This work moves beyond association to provide direct experimental evidence that gut microbial H₂S is a contributing factor of metabolic dysfunction. It delineates a mechanistic axis linking a specific gut microbial metabolic product to the reprogramming of host hepatic metabolism, offering additional insights into the microbiome’s role in metabolic disease and highlighting potential targets for microbiome-based therapeutics.

## Materials and methods

### Public database for intestinal metagenomic analysis

To elucidate the differences in intestinal microbiota between individuals with glycolipid metabolism disorders and healthy controls, we summarized data from two cohorts: one comprising non-obese (NOB) and obese (OB) populations, and the other consisting of individuals without glucose metabolism disorders (NGMD) and with it (GMD). The NOB-OB data was obtained from a curated database of human gut metagenomes GMrepo v2 using the keywords “health” and “obesity”, including 924 NOB individuals and 465 OB individuals, all of whom were not labeled as antibiotic positive. The distinction between “healthy” and “obese” populations was made by GMrepo related technicians based on baseline information or instructions from the original article authors [19]. The metagenomic data for microbial gene analysis in the cohort with glucose metabolism disorders was obtained from PRJNA686835 [20] and CNP0000175 [21], encompassing 79 NGMD individuals and 112 individuals with GMD, all of whom did not use antibiotics. The population with normal glucose metabolism and those with glucose metabolism disorders were determined by the original author based on fasting blood glucose or oral glucose tolerance tests. For detailed genomic information, please refer to Supplementary Data Sheet 1 and Sheet  2.

Perform quality control on the raw files to obtain high-quality sequences. Remove the adapter sequences from the raw sequences using Cutadapt. Filter low-quality sequences using Fastp, and remove polymerase chain reaction (PCR) duplicate sequences using Fastuniq. Align the raw sequences to the human reference genome (*Homo sapiens*, hg38) using Bowtie2 to remove human-derived sequences and retain only high-quality non-human-derived sequences. Subsequently, use the high-quality sequences for functional gene analysis. For metagenome assembly, generate contigs from the high-quality sequences using Megahit. The assembled contigs undergo functional gene prediction using Prodigal, and a non-redundant gene set is constructed using CD-HIT. Estimate gene relative abundance using Salmon, perform functional annotation using eggNOG-mapper, and map the sequences to the KEGG database to obtain gene function and metabolic pathway information. In addition, we further screened genes of “Bacteria” origin in eggNOG for enrichment analysis.

### Bacterial culture

*D. desulfuricans* was isolated from the intestinal tract of mice. The organism was cultured in an anaerobic environment at 30°C for 7 days, using a medium consisting of 0.5 g/L KH_2_PO_4_, 1.0 g/L NH_4_Cl, 1.0 g/L Na_2_SO_4_, 0.05 g/L CaCl_2_, 2.0 g/L MgCl_2_·6H_2_O, 1.0 g/L yeast extract, 0.1 g/L ascorbic acid, 0.1 g/L sodium thioglycolate, and 1.1 g/L DL-lactic acid sodium salt.

### Engineered H_2_S-producing *E. coli*

The dissimilatory sulfite reductase *dsrAB* was amplified from the genomic DNA of *D. desulfuricans* by PCR with homology arms and subsequently assembled into the pETDuet vector using a DNA Assembly Mix Ultra (EG24204S; BestEnzymes Biotech) according to the manufacturer’s instructions. In parallel, the thiosulfate reductase operon *phsABC* was amplified from the genomic DNA of *Salmonella typhimurium ATCC 14028* and similarly subcloned into pETDuet. Primers and expansion procedures can be found in the Supplementary Data Sheet 3 and Sheet  4. Engineered *E. coli* was subsequently induced and cultured in lead acetate medium to evaluate its capacity to produce hydrogen sulfide.

### Animals and treatments

Six-week-old male C57BL/6J mice, specific-pathogen-free (SPF), were used in the animal experimental procedures of this study. These mice were obtained from Hunan Sleek Jingda (Changsha, China) and housed in cages with metal grid covers under stringent sterile conditions, with controlled temperature (25 ± 5°C), humidity (60%–70%), and a 12-h light/dark cycle. The mice had unrestricted access to potable water and standard rodent food. All animal experiments were conducted in accordance with the National Institutes of Health Guide for the Care and Use of Laboratory Animals and approved by the Institutional Ethics Committee for Animal Procedures at Central South University (CSU-2024-0315).

Six independent protocols (n = 5 per group) were established for this experiment, each receiving standard feed. Unless otherwise specified, gavage administration was performed at a volume of 0.1 mL/10 g body weight. First: Mice in the control group (ND) were provided with a normal diet, while mice in the experimental group (HFD) received 60% high-fat diet (D12492; Xiao Shu You Tai Beijing Biotechnology Co., Ltd) (Fig. 1C). Second: Both the control group (CD) and the experimental group (DSV) underwent daily gavage and twice-weekly enema. The CD group received the vehicle phosphate-buffered saline (PBS) alone, whereas the DSV group received 1 × 10^8^ colony-forming units (CFU)/mL of *D. desulfuricans* via the same routes (Fig. 2A). Third: Both the control group (CD) and the experimental group (*phsABC*) underwent daily gavage and twice-weekly enema. The CD group received the vehicle PBS alone, whereas the *phsABC* group received 1 × 10^9^ CFU/mL of engineered H_2_S-producing *E. coli* via the same routes (Fig. 2D). Fourth: Mice in the control group (CD) were provided with a normal diet and water, while the experimental group (NaHS) received 5 mmol/L NaHS (O0656; TCI Shanghai) mixed with drinking water daily for the intervention (Fig. 2G). Fifth: Mice in the control group (CD) were provided with a normal diet and water and received intraperitoneal injections of vehicle (three times per week). Mice in the NaHS group received 5 mmol/L NaHS in drinking water daily, along with intraperitoneal injections of vehicle (three times per week). Mice in the OEA group received 5 mmol/L NaHS in drinking water daily, and were additionally given intraperitoneal injections of the PPARα agonist N-Oleoylethanolamine (OEA) (N859459; Macklin) at a dose of 5 mg/kg (three times per week) (Fig. 4J). Sixth: From weeks 1 to 8, the control group (CD) mice received a standard diet and drinking water, while the other mice were administered 5 mmol/L NaHS in their drinking water. In the ninth week, the NaHS-treated mice were randomly divided into two groups. From weeks 9 to 11, the control group (CD) mice continued to receive a normal diet and drinking water. One group (NaHS) maintained the 5 mmol/L NaHS intervention, while the other group (C_7_H_5_BiO_4_) had an additional 20 mmol/L of bismuth subsalicylate (B107666; aladdin) incorporated into their drinking water (Fig. 5F).

**Figure 1:**
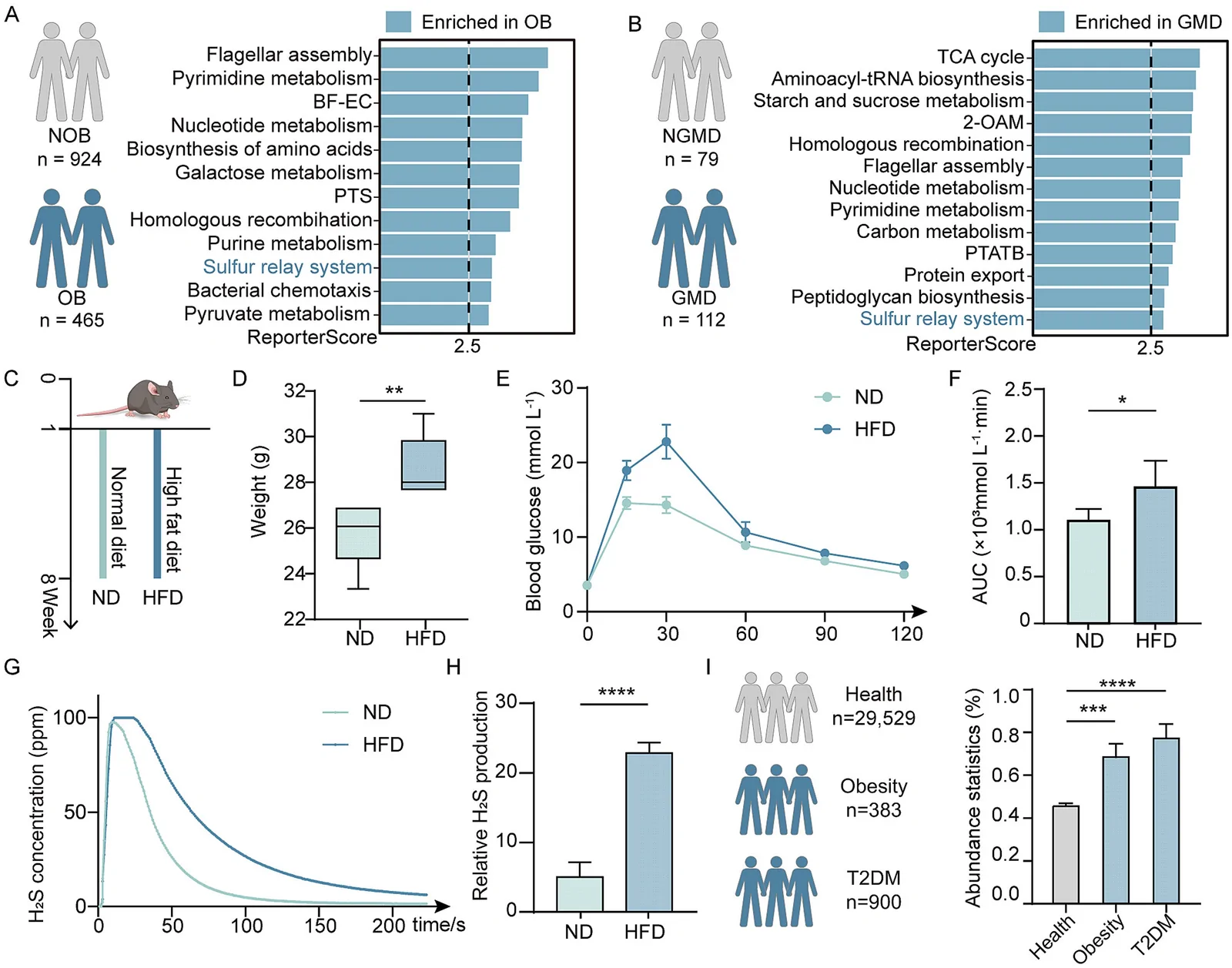
Disorders of glycolipid metabolism are linked to abnormal sulfur metabolism in feces. (A) The analysis includes non-obese (NOB) and obese (OB) population cohorts, highlighting the enrichment of fecal differential genes in the obese group (PTS: Phosphotransferase system). (B) This section examines cohorts with non-glucose metabolism disorders (NGMD) and those with glucose metabolism disorders (GMD), emphasizing the enrichment of fecal differential genes in the glucose metabolism disorder population (PTATB: Phenylalanine, tyrosine and tryptophan biosynthesis). (C) Intervention on mice with high-fat diet (HFD) and normal diet (ND) for 7 weeks. (D) The body weight of mice is compared between the ND group and HFD group. (E) OGTT is conducted for mice in both the ND group and the HFD group. (F) The cumulative area under the curve (AUC) of OGTT. (G) H_2_S concentrations in the headspace of fecal fermentation. (H) The relative content of hydrogen sulfide in fecal fermentation liquid. (I) Population data are collected from the GMrepo website. The abundance statistics of fecal *Desulfovibrio* is assessed in health, obesity, and T2DM populations. ^*^P < .05, ^**^P < .01, ^***^P < .001, ^****^P < .0001.

**Figure 2:**
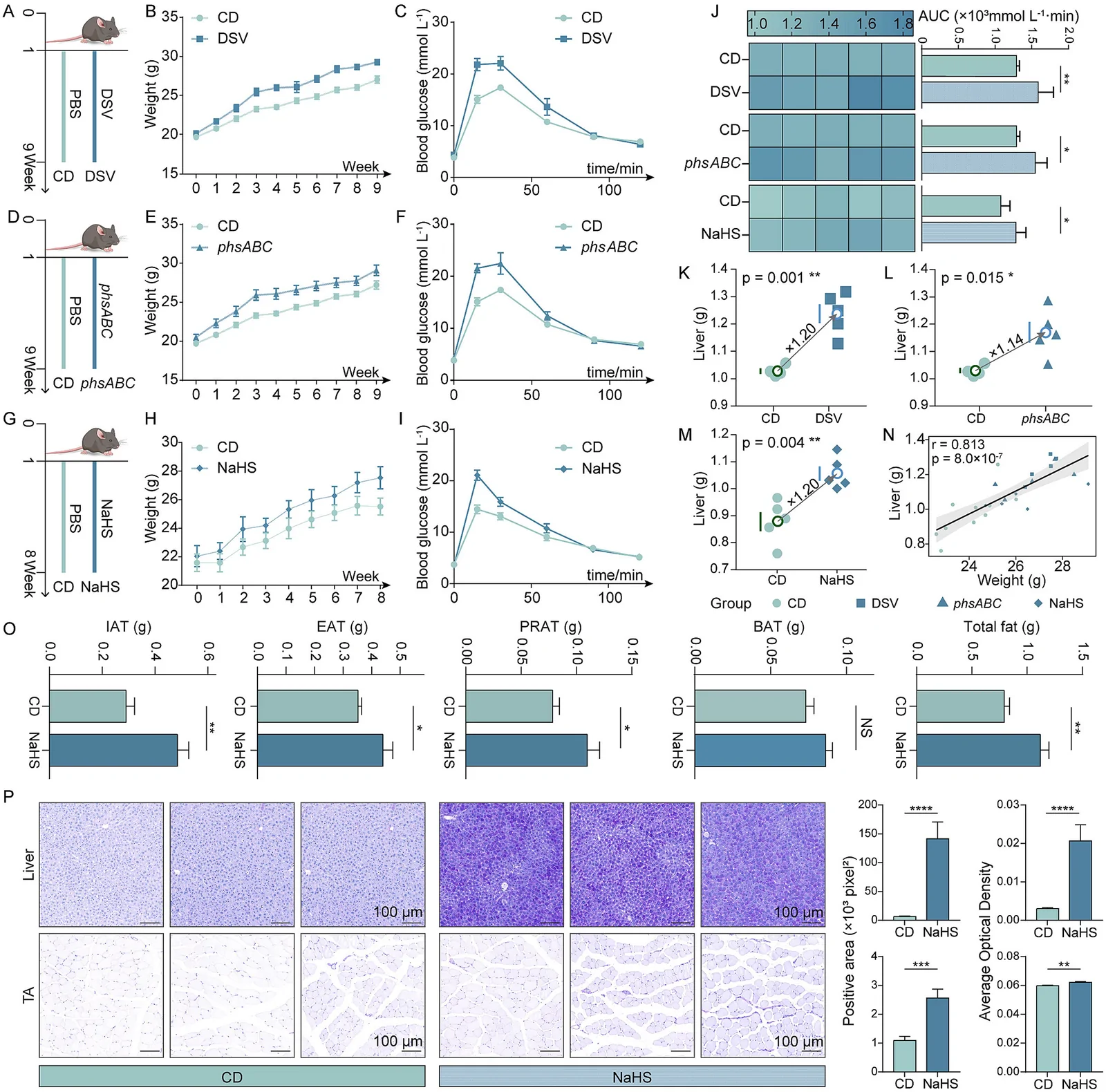
SRB causes disorders in glycolipid metabolism by generating H_2_S. (A) Intervention of mice with PBS (CD) and *D. desulfuricans* (*DSV*) for 8 weeks. (B) Trends of body weight changes in mice of the CD group and the *DSV* group. (C) OGTT is conducted for mice in both the CD group and the *DSV* group. (D) PBS (CD) and engineered H_2_S-producing *E. coli* (*phs*ABC) was administered to intervene in mice for 8 weeks. (E) Trends in body weight changes of mice in the CD group and the *phs*ABC group. (F) OGTT is conducted for mice in both the CD group and the *phs*ABC group. (G) PBS (CD) and H_2_S donor NaHS (NaHS) were administered by watering to intervene in mice for 7 weeks. (H) Trends in body weight changes of mice in the CD group and the NaHS group. (I) OGTT is conducted for mice in both the CD group and the NaHS group. (J) The cumulative area under the curve (AUC) of OGTT. (K) The liver weight of mice is compared between the CD group and *DSV* group. (L) The liver weight of mice is compared between the CD group and *phs*ABC group. (M) The liver weight of mice is compared between the CD group and NaHS. (N) Linear regression of body weight and liver weight in group. (O) Comparison of adipose tissue weight between the CD group and the NaHS group. (P) Comparison of liver glycogen staining pathological sections, positive areas, and average optical density between the CD group and the NaHS group. ^*^P < .05, ^**^P < .01, ^***^P < .001, ^****^P < .0001.

### Weight, fasting blood glucose, and glucose tolerance monitoring

The body weight of all mice was recorded weekly. Fasting blood glucose levels and glucose tolerance were assessed. For the oral glucose tolerance test (OGTT), mice were fasted for 12 h with free access to water. After measuring fasting body weight and blood glucose levels, a 20% glucose solution (0.01 mL/g body weight) was administered via intragastric gavage, and blood glucose concentrations were measured at 15, 30, 60, 90, and 120 min post-gavage.

### Tissue collection and pathological analysis

After a 12-h fast, mice were euthanized by cervical dislocation. Liver, adipose tissues—subcutaneous inguinal adipose tissue (IAT), epididymal adipose tissue (EAT), perirenal adipose tissue (PRAT), brown adipose tissue (BAT), and colon were collected and weighed. Liver specimens were fixed in neutral buffered formalin (4% paraformaldehyde) for H&E staining; additional liver and muscle specimens were fixed in the same fixative for PAS staining; and colon specimens were fixed in the same fixative for immunofluorescence staining of ZO-1 and Occludin.

### RNA-seq and transcriptomic analysis of liver tissues

Total RNA was extracted from tissues using the MJZol total RNA extraction kit, with genomic DNA and proteins subsequently removed. RNA integrity and concentration were assessed using the Agilent 5300 and NanoDrop 2000, respectively. Polyadenylated mRNA was enriched using oligo (dT) magnetic beads and chemically fragmented. Subsequent steps involved reverse transcription to synthesize cDNA, construction of flat ends, and ligation of adaptors. The target cDNA fragment, ranging from 200 to 300 bp, was selected using Biowest agarose, amplified with DNA polymerase, and purified to yield the final library. Double-ended sequencing was performed by Shanghai Meiji Biomedical Technology Co., Ltd. using the DNBSEQ platform. The original sequencing data underwent quality control using Fastp. The clean reads were then aligned to the reference genome (GRCm39) using Hisat2. The count of clean read segments was determined using RSEM.

### Quantitative PCR

*PPARα* mRNA expression in liver tissues – Total RNA was extracted from liver tissues using TRIzol reagent (SK1312; Sango) and reverse-transcribed into cDNA with the AMV First Strand cDNA Synthesis Kit (SK2445, Sango). Primers targeting mouse *PPARα* and *β-actin* (internal control) were designed based on conserved regions (Supplementary Data Sheet 5). Real-time PCR was conducted using SuperStar Universal SYBR Master Mix (CW3360; CWbio) on a SLAN-96S system. Relative *PPARα* expression was calculated by the 2^−ΔΔCt^ method, with *β-actin* as the endogenous reference.

Quantification of fecal *Desulfovibrio* 16S and *phsABC* genes – Genomic DNA was extracted from fecal samples using the Universal Genomic DNA Extraction Kit (D2100; Solarbio). Primers targeting *Desulfovibrio* 16S rRNA, *phsABC*, and total bacterial 16S rRNA were designed (Supplementary Data Sheet 5). Real-time PCR was performed as described above. The relative abundances of *Desulfovibrio* 16S and *phsABC* were determined using the 2^−ΔCt^ method, with total bacterial 16S rRNA as the reference.

### Cell culture and treatment

HepG2 cell lines were obtained from Procell Life Science & Technology Co., Ltd. (Wuhan, China). Cells were maintained in Dulbecco’s modified Eagle’s medium (DMEM; Gibco) containing 4.5 g/L glucose, supplemented with 10% fetal bovine serum (2 342 332, VivaCell BIOSCIENCES) and 1% penicillin/streptomycin (RP-RS1021, Report) at 37°C in a humidified atmosphere containing 5% CO₂. In certain experiments, HepG2 cells were treated with NaHS (O0656; TCI Shanghai) for 24 h, with NaHS administered every 6 h. According to experimental requirements, cells were pretreated with fenofibrate (KM12420, KKL Med Inc., 100 μM) or N-Oleoylethanolamide (N859459, Macklin, 100 μM) for 30 min prior to NaHS exposure, or left untreated as controls. Control cells received an equal volume of the corresponding vehicle.

### Protein extraction and Western blotting

Cells and tissues were washed twice with PBS, lysed in RIPA buffer (Beyotime) containing protease inhibitor cocktail, and incubated on ice for 30 min. After centrifugation at 12000 rpm for 10 min at 4°C, supernatants were collected and denatured in 5× loading buffer at 95°C for 5 min. Equal amounts of protein were resolved on MOPS Running Buffer (SW156-12B; Seven/Abcells), transferred to polyvinylidene fluoride membranes by wet transfer, and blocked with 5% non-fat milk in TBST for 1 h at room temperature. Membranes were then incubated overnight at 4°C with primary antibodies. After three 10-min washes with tris-buffered saline with tween-20 (TBST), membranes were incubated with horseradish peroxidase-conjugated secondary antibodies for 1 h at room temperature. Following three additional 10-min TBST washes, signals were visualized using enhanced chemiluminescence. GAPDH served as the loading control. Band intensities were quantified with Bio-Rad and ImageJ software.

### Biochemical index detection

At the end of the experimental period, all mice were fasted overnight and anesthetized. Blood samples were collected from the retro-orbital plexus and allowed to clot at ice temperature for 30 min. Serum was separated by centrifugation at 12 000 rpm for 10 min at 4°C, which was immediately subjected to measurement of endogenous H₂S levels using a commercial assay kit (A146-1-1; Nanjing Jiancheng Bioengineering Institute). Serum levels of IL-6 and TNF-α were quantified using commercial mouse-specific ELISA kits (M206427-102a; M260415-102a; NEOBIOSCIENCE).

Fecal H_2_S production was assessed by anaerobic fermentation under N_2_/H_2_/CO_2_ (80:10:10, v/v/v). For liquid-phase analysis, 0.1 mL of fecal suspension was inoculated into 10 mL of Postgate medium(add 0.5 g/L FeSO_4_·7H_2_O) and incubated at 37°C and 200 rpm for 12 h. Fermentation liquid was transferred to a 96-well plate (200 μL/well, three technical replicates), and grayscale intensity was quantified using ImageJ as a relative measure of H_2_S production. For headspace analysis, 1 mL of fecal suspension was inoculated into 200 mL of pre-reduced Postgate medium and incubated for 24 h under the same temperature and shaking conditions. Headspace H_2_S was quantified using an H_2_S gas detection module(XS-BC-H_2_S; X-gas Intelligent Technology) with high-purity N_2_ as the carrier gas at a flow rate of 0.6 L/min.

After cell intervention, the prepared cell suspension was collected and centrifuged at 1000 rpm for 10 min. The supernatant was discarded, and the cell pellet was retained. The cells were washed twice with isotonic PBS, each wash followed by centrifugation at 1000 rpm for 10 min, and the supernatant was removed. The final cell pellet was resuspended in 0.2 mL PBS and homogenized by ultrasonication on an ice-water bath. The resulting homogenate was used to determine glucose levels using a Glucose kit based on the glucose oxidase method (A154-1-1; Nanjing Jiancheng Bioengineering Institute).

### Statistics

All data were tested for normality using the Shapiro–Wilk test and for variance using Levene’s test. Two-group comparisons were performed using a two-sided unpaired Student’s *t-*test or Mann–Whitney U test, as appropriate. Comparisons among three groups were performed using one-way ANOVA with multiple comparison tests. Unless otherwise specified, all images were plotted using the ggplot2 package. Data were presented as mean ± SEM. Differential gene expression analysis was performed using DESeq2, employing a cutoff of *padj* < 0.05 and |log2FoldChange| ≥ 1. Gene enrichment analysis was performed using the R package “ReporterScore”. The Benjamini-Hochberg method was used for multiple hypothesis testing correction in both the differential analysis of KO genes and the evaluation of pathway ReporterScores. Gene functions and coding proteins were referenced from the KEGG database (https://www.kegg.jp/kegg/).

## Results

### Fecal desulfovibrio abundance and H₂S production are elevated in glucose and lipid metabolism disorders

We analyzed publicly available gut metagenomic datasets from cohorts characterized by obesity and impaired glucose metabolism, including a total of 1580 runs as detailed in the methods section. Our analysis revealed a significant enrichment of genes involved in flagellar assembly, pyrimidine metabolism, *E. coli* biofilm formation (BF-EC), and nucleotide metabolism in the feces of obese individuals (Fig. 1A). In the population with glucose metabolism disorders, genes related to the citrate cycle, aminoacyl-tRNA biosynthesis, starch and sucrose metabolism, and 2-oxocarboxylic acid metabolism (2-OAM) were enriched (Fig. 1B). Notably, the gene associated with the sulfur relay system was enriched across both cohorts (Fig. 1A and B). Subsequently, statistical analysis of genes involved in sulfur cycle revealed that genes associated with sulfur transport and H₂S production in fecal microbiota were more abundant in the cohorts with obesity and glucose metabolism disorders (Fig. S1). For instance, the abundance of genes encoding *phsA*, *phsC*, *dsrA*, and *dsrB* was significantly increased in the cohorts with obesity (Fig. S1A–E), while *phsA*, *phsB*, *phsC*, *dsrA*, and *dsrB* showed a trend toward higher abundance in the cohorts with glucose metabolism disorders (Fig. S1K–O).

To further investigate whether the enrichment of the sulfur cycle gene contributes to increased H_2_S production, we established mouse models exhibiting obesity and glucose metabolism disorders (Fig. 1C). Following a 7-week high-fat diet intervention, the HFD group exhibited significantly higher body weights (Fig. 1D), accompanied by impaired oral glucose tolerance (Fig. 1E and F). Anaerobic fermentation revealed that fecal samples from the HFD group produced significantly higher concentrations of H_2_S in both the flask headspace and the fermentation liquid compared to the ND group (Fig. 1G and H). To identify the key taxa responsible for H_2_S production, we queried the GMrepo database, which revealed that the relative abundance of *Desulfovibrio* was significantly higher in obese and type 2 diabetic populations compared to healthy individuals (Fig. 1I). These findings indicate a potential association between *Desulfovibrio* and *Desulfovibrio*-derived H_2_S and glycolipid metabolic dysfunction.

### *D. desulfuricans*, engineered H_2_S-producing *E. coli*, and H_2_S donor all contribute to weight gain and glucose metabolism disorders

We first administered *D. desulfuricans* to mice for 8 weeks (Fig. 2A). Fecal microbiota analysis showed that the relative abundance of *Desulfovibrio* was higher in the DSV group than CD group (Fig. S2F). Moreover, serum H₂S levels were markedly elevated in the DSV group (Fig. S2J). Consistent with these microbial and metabolic changes, *D. desulfuricans*-treated mice exhibited significant weight gain (Fig. 2B) and impaired oral glucose tolerance (Fig. 2C and J). To identify the underlying molecular mechanism, we heterologously expressed the *dsrAB* (Fig. S2D) from *D. desulfuricans*. However, the recombinant protein failed to exhibit functional sulfate reductase activity (Fig. S2A). Consequently, we employed the *phsABC* (Fig. S2E) from *S. typhimurium ATCC14028* for heterologous expression, which successfully produced H_2_S as a surrogate tool for local H_2_S generation (Fig. S2B). We administered engineered H_2_S-producing *E. coli* to mice for 8 weeks (Fig. 2D). Fecal microbiota analysis revealed that the fecal *phsABC* relative abundance was significantly elevated in the *phsABC* group compared to the CD group (Fig. S2G). Consistent with this microbial colonization, serum H₂S levels were also markedly increased in the *phsABC* group (Fig. S2K). Consequently, these mice developed significant weight gain (Fig. 2E) and impaired oral glucose tolerance (Fig. 2F and J). To confirm the role of the metabolic product, we administered the H_2_S donor NaHS to mice via drinking water for 7 weeks (Fig. 2G). Serum H₂S levels were significantly elevated in the NaHS-treated group (Fig. S2L). Consistent with our previous findings, the NaHS-treated group exhibited a significant increase in body weight (Fig. 2H) and impaired oral glucose tolerance (Fig. 2I and J). Taken together, these findings support a contributory role of increased H_2_S exposure in weight gain and impaired glucose metabolism in mice.

### H₂S promotes hepatic and skeletal muscle glycogen accumulation and systemic fat deposition

We dissected and weighed several organs and tissues related to glucose and lipid metabolism, including the liver, inguinal (IAT), epididymal (EAT), perirenal (PRAT), and brown adipose tissue (BAT). Liver mass was significantly increased in the H_2_S- accumulation group (Fig. 2K–M). Furthermore, the weights of the IAT, EAT, and PRAT were markedly elevated in the NaHS group (Fig. 2O) and positively correlated with total body weight (Fig. 2O). Notably, liver mass exhibited the most significant positive correlation with total body weight among the tissues analyzed (Fig. 2N and Fig. S2N). Histological analysis of hepatic glycogen via PAS staining revealed significant increases in both the stain-positive area and mean optical density in the NaHS group (Fig. 2P). Parallel increases in glycogen content were observed in the tibialis anterior (TA) muscle (Fig. 2P). In addition, H&E staining of liver sections showed mild focal inflammatory changes in both the H₂S-producing microbial group and the NaHS group (Fig. S3A–C), although serum levels of IL-6 and TNF-α showed no significant differences compared to controls (Fig. S2H and I). Furthermore, to assess whether H_2_S-related interventions were associated with alterations in intestinal barrier integrity, we performed immunofluorescence staining of the colonic tight junction proteins ZO-1 and Occludin (Fig. S3D–F). Notably, both ZO-1 and Occludin exhibited reductions in the H₂S-producing microbial intervention group and the NaHS group compared to CD group (Fig. S3G and H). Collectively, these findings suggest that hydrogen sulfide contributes to the accumulation of both glycogen and lipids.

### H_2_S modulates hepatic transcriptional profiles

We conducted transcriptomic profiling via RNA-seq on liver tissue obtained from the experimental mice (Fig. 2G). Principal component analysis (PCA) revealed a distinct separation of global gene expression profiles between the CD and NaHS-treated groups (Fig. 3A). Specifically, we identified 1869 and 2146 specifically expressed genes in the CD and NaHS groups, respectively (Fig. 3B). To investigate whether exposure induced de novo gene activation or transcriptional silencing, we performed differential expression tests on the genes uniquely present in each group. This analysis revealed no significant differences in the expression levels of these specific genes between the two groups (Fig. 3B). Collectively, these findings suggest that H_2_S promotes glycolipid metabolic dysfunction primarily through the up-regulation or down-regulation of key regulatory genes rather than large-scale gene activation or silencing.

**Figure 3:**
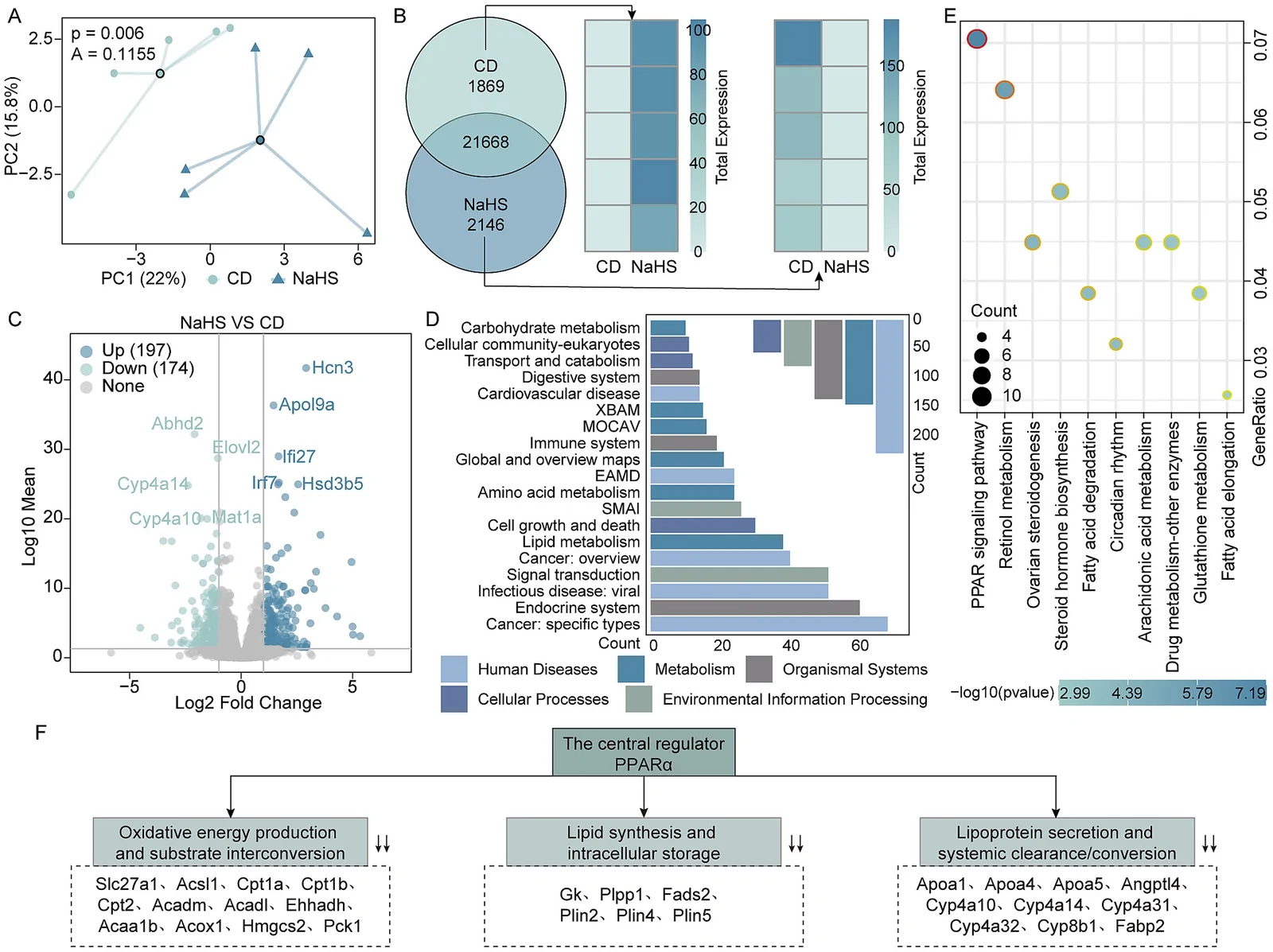
H_2_S influences transcriptional levels in the liver. (A) PCA analysis of liver genes. (B) The expression of specific genes in each group in the CD group and the NaHS group. (C) Differentially expressed genes of CD group and the NaHS group. (D) Statistical analysis of differentially expressed genes in the KEGG pathway. (E) KEGG pathway enrichment of DEGs. (F) Functional classification of differentially expressed genes enriched in the PPAR signaling pathway.

We performed differential expression analysis to compare the hepatic transcriptomes of the CD and NaHS groups. The results revealed 197 significantly upregulated transcripts, including *Hcn3*, *Apol9a*, *Ifi27*, *Irf7*, and *Hsd3b5*, and 174 significantly downregulated transcripts, such as *Abhd2*, *Elovl2*, *Cyp4a14*, *Cyp4a10*, and *Mat1*, in the NaHS group (Fig. 3C). Functional annotation categorized the majority of these differentially expressed genes (DEGs) into pathways related to human diseases, metabolism, and organismal systems, specifically focusing on endocrine function and lipid metabolism (Fig. 3D). Pathway enrichment analysis further demonstrated that DEGs were notably enriched in the PPAR signaling, retinol metabolism pathways, among others (Fig. 3E). We found that the PPAR signaling pathway harbored the largest number of differentially expressed genes and exhibited the highest GeneRatio among all enriched pathways (Fig. 3E). Within this pathway, the central regulator PPARα was significantly downregulated. Concomitantly, downstream genes involved in oxidative energy production and substrate interconversion, lipid synthesis and intracellular storage, and lipoprotein secretion and systemic clearance/conversion were also markedly reduced (Fig. 3F).

### H_2_S downregulates key regulatory genes involved the PPAR signaling pathways

Gene Set Enrichment Analysis (GSEA) confirmed a downward trend in the transcription of gene sets associated with PPAR signaling in the livers of NaHS-treated mice (Fig. 4A). Consistent with this finding, we further quantified the expression of the central regulator *PPARα*. At the transcriptional level, hepatic *PPARα* mRNA transcription was significantly reduced in the NaHS-treated mice compared to controls (Fig. 4B). Similarly, at the protein level, PPARα expression in the liver was also significantly decreased following NaHS treatment (Fig. 4C).

**Figure 4:**
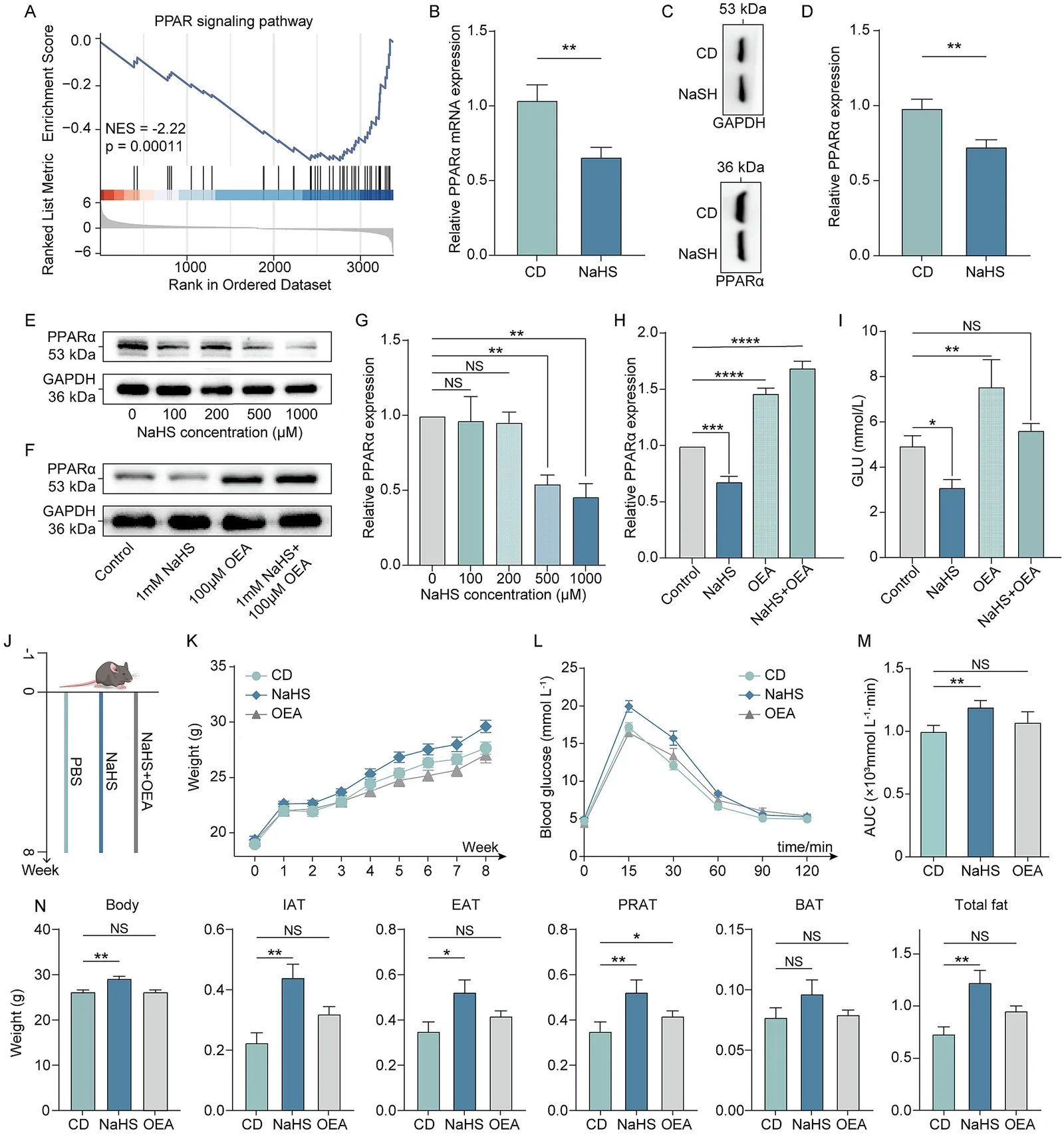
H_2_S downregulates key regulatory genes involved in the PPAR signaling pathway. (A) GSEA of liver transcriptomes from NaHS-treated versus control mice, showing significant negative enrichment of the PPAR signaling pathway gene set. (B) Hepatic PPARα mRNA expression in control and NaHS-treated mice determined by quantitative PCR. (C) Representative Western blot bands of PPARα protein in mouse liver tissues from control and NaHS groups. (D) Densitometric quantification of PPARα protein expression in mouse liver tissues, normalized to GAPDH. (E) Representative Western blot of PPARα protein in HepG2 cells treated with increasing concentrations of NaHS (0, 100, 200, 500, 1000 μM). (F) Representative Western blot of PPARα protein in HepG2 cells treated with NaHS (1000 μM) with or without the PPARα agonist OEA (100 μM). (G) Densitometric quantification of PPARα protein expression in HepG2 cells following gradient NaHS treatment, normalized to GAPDH. (H) Densitometric quantification of PPARα protein expression in HepG2 cells after NaHS and/or OEA treatment. (I) Cellular glucose uptake capacity in HepG2 cells measured by a glucose oxidase-based assay following NaHS and/or OEA treatment. (J) The intervention process involving the PPARα agonist OEA was outlined. (K) Trends of body weight changes in mice of the CD group, NaHS group, and OEA group. (L) OGTT were conducted on mice in the CD group, NaHS group, and OEA group. (M) The cumulative area under the curve (AUC) of OGTT. (N) Comparison of tissue weight among CD group, NaHS group, and OEA group. ^*^P < .05, ^**^P < .01, ^***^P < .001, ^****^P < .0001.

To further elucidate the functional role of H₂S in modulating the PPAR signaling pathway, we investigated the interaction between H₂S and PPARα protein expression in vitro (Fig. 4E). In HepG2 cells, treatment with 1000 μM NaHS induced the most pronounced decrease in PPARα protein expression, with a reduction of ~50% (Fig. 4G). To determine whether PPARα activation could counteract this NaHS-induced decrease, we treated HepG2 cells with the PPARα agonist OEA (Fig. 4F). Administration of the PPARα agonist OEA significantly upregulated PPARα protein expression. Notably, co-treatment of HepG2 cells with NaHS and OEA restored PPARα expression to levels significantly higher than those observed in both the control group and the NaHS-alone group (Fig. 4H). In addition, we assessed glucose uptake capacity in HepG2 cells. Treatment with 1000 μM NaHS significantly reduced glucose uptake, whereas OEA treatment markedly enhanced it. Notably, co-treatment with NaHS and OEA restored glucose uptake to normal levels (Fig. 4I). When another PPARα agonist, fenofibrate (INN), was used under the same experimental conditions, PPARα expression exhibited a similar trend (Fig. S4B and C).

To assess the in vivo effect of PPARα activation, we administered OEA to mice as an intervention (Fig. 4J). Consistently with the in vitro findings, NaHS treatment alone significantly reduced hepatic PPARα protein expression in mice, whereas co-treatment with OEA rescued this reduction (Fig. S4D and F). In parallel experiments, mice co-treated with NaHS and OEA exhibited attenuated NaHS-induced weight gain, counteracting the effect induced by NaHS alone (Fig. 4K). Moreover, the weights of various adipose depots, including IAT, EAT, BAT, and total fat were restored to levels comparable to those of the control group (Fig. 4N). Furthermore, glucose tolerance in the co-treated mice, as assessed by the OGTT, returned to normal levels (Fig. 4L), and the AUC was not significantly different from that of the control group (Fig. 4M).

### H_2_S downregulates regulatory genes within the lipid metabolism pathways

To further elucidate the impact of H_2_S on lipid homeostasis, we sub-categorized the DEGs within the organismal systems and metabolism pathways. Aside from the Cyp gene family, the majority of these genes were associated with lipid metabolism (Fig. 5A). GSEA of lipid metabolism-related genes revealed that the gene sets corresponding to biosynthesis of unsaturated fatty acids, fatty acid elongation, fatty acid degradation, and fatty acid metabolism were significantly downregulated (Fig. S4A). Protein–protein interaction (PPI) network analysis (Fig. 5B) identified three central nodes involved in fatty acid metabolism: *Acot1*, *Acot2*, and *Ehhadh* (Fig. 5C). These key genes are cross-listed in both the lipid metabolism and endocrine system pathways (Fig. 5C). In our earlier analysis, we noted that the DEGs were enriched in the fatty acid degradation and elongation pathways (Fig. 3E). Furthermore, *Acot1*, *Acot2*, and *Ehhadh* are implicated in both the fatty acid degradation and elongation processes (Fig. 5D and E).

**Figure 5:**
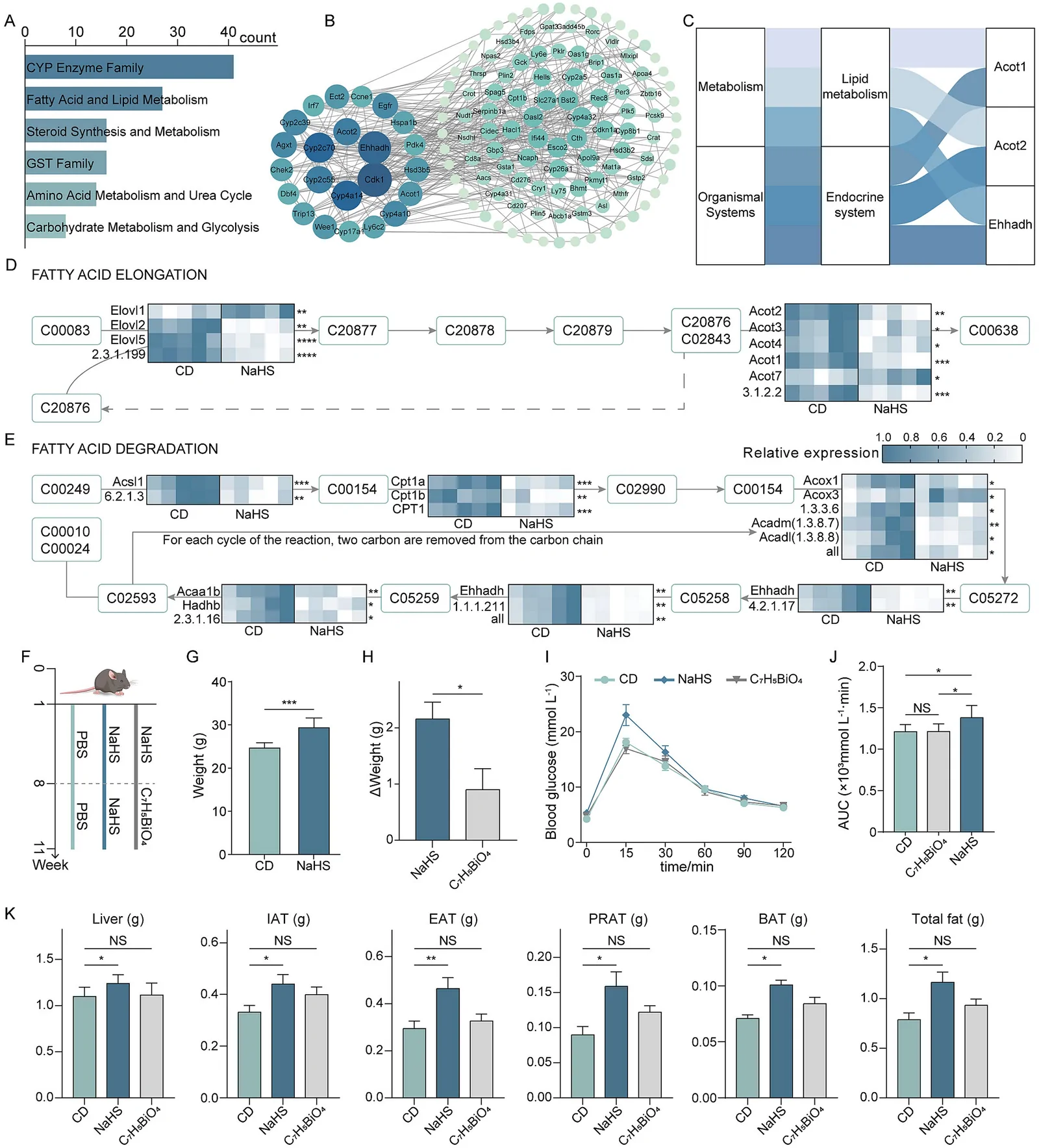
H_2_S downregulates genes related to glycolipid metabolism pathways. (A) Differential gene classification was carried out. (B) PPI network analysis was performed on differentially expressed genes. (C) Key genes related to fatty acid and lipid metabolism were identified. (D) The expression of genes in the fatty acid elongation pathway was assessed in both the CD group and the NaHS group. (E) In the fatty acid degradation pathway, gene expression was evaluated in the CD group and the NaHS group. (F) The intervention process involving the H_2_S adsorbent C_7_H_5_BiO_4_ was outlined. (G) Changes in weight following NaHS intervention were recorded. (H) Weight gain was measured in mice from the NaHS group and the C_7_H_5_BiO_4_ group between 8 and 11 weeks. (I) OGTT were conducted on mice in the CD group, NaHS group, and C_7_H_5_BiO_4_ group. (J) The cumulative area under the curve (AUC) of OGTT. (K) Comparison of tissue weight among CD group, NaHS group, and C_7_H_5_BiO_4_ group. ^*^P < .05, ^**^P < .01, ^***^P < .001, ^****^P < .0001.

We conducted a statistical analysis of the genes involved in these pathways. In the fatty acid elongation pathway, the expression levels of genes related to very long-chain 3-oxoacyl-CoA synthase (2.3.1.199) and long-chain fatty acyl-CoA hydrolase (3.1.2.2) significantly decreased. Notably, the expression of the Acot1 and Acot2 genes also decreased significantly (Fig. 5D). In the fatty acid degradation pathway, there was a significant reduction in the expression of genes related to long-chain fatty acid-CoA ligase (6.2.1.3), CPT1, acyl-CoA oxidase (1.3.3.6), medium-chain acyl-CoA dehydrogenase (1.3.8.7), long-chain-acyl-CoA dehydrogenase (1.3.8.8), enoyl-CoA hydratase (4.2.1.17), 3-hydroxyacyl-CoA dehydrogenase (*Ehhadh*), long-chain-3-hydroxyacyl-CoA dehydrogenase (1.1.1.211), and acetyl-CoA C-acyltransferase (2.3.1.16) (Fig. 5E).

### The H_2_S adsorbent bismuth subsalicylate (C_7_H_5_BiO_4_) mitigates established NaHS-associated glycolipid metabolic dysfunction

To evaluate the therapeutic potential of H₂S sequestration, we pre-established a mouse model of metabolic dysfunction induced by exogenous NaHS intervention (Fig. 5F and G). Measurement of serum H₂S concentrations confirmed that C_7_H_5_BiO_4_ treatment significantly reduced the elevated H₂S levels induced by NaHS intervention (Fig. S2M). Compared to the NaHS-only group, mice treated with the H₂S adsorbent C_7_H_5_BiO_4_ exhibited significantly attenuated weight gain (Fig. 5H). Furthermore, glucose excursion during OGTT was normalized, with results returning to levels comparable to the control group (Fig. 5I and J). Systematic dissection and weighing of metabolic tissues revealed that intervention with the H₂S adsorbent led to a significant reduction in the weights of liver, IAT, EAT, PRAT, BAT and the total fat (Fig. 5K). Hepatic PAS staining further demonstrated that glycogen accumulation was normalized, as the stain-positive area and mean optical density in the C_7_H_5_BiO_4_-treated group were not significantly different from those of the control group (Fig. S4H). Moreover, PPARα expression was significantly restored relative to the NaHS-only group (Fig. S4E and G). These findings collectively indicate that H_2_S sequestration mitigates established H_2_S-associated metabolic abnormalities.

## Discussion

This study provides experimental evidence in mouse models supporting a contributory role of gut microbiota-derived H₂S in the development of obesity and glucose metabolism disorders. By integrating human metagenomic analyses with systematic intervention experiments in mouse models and in vitro cellular assays, our mouse-based experiments suggest that H₂S produced by gut microbiota may function as more than a mere biomarker, potentially playing a role in the progression of metabolic disorders in these models. In human cohorts with obesity and glucose metabolism disorders, we observed a notable enrichment of microbial genes involved in sulfur transport and H₂S production. Through separate administration of H₂S-producing bacteria, a functional H₂S synthase, and H₂S itself, we successfully induced weight gain and impaired glucose tolerance in mice, suggesting that gut-derived H₂S participates in metabolic dysfunction. These findings are consistent with prior reports of increased *Desulfovibrio* abundance in individuals with obesity [14], type 2 diabetes [22], and gestational diabetes [23].

A notable contribution of this study is the attempt to establish a possible causal chain connecting microbial H₂S production to systemic metabolic changes in mouse models. Previous studies have noted the association between *Desulfovibrio* abundance and metabolic disorders but have not conclusively determined the causative role of H₂S. Our research adds to this body of evidence by demonstrating that *D. desulfuricans* and the functional enzyme *phsABC*, both capable of H₂S synthesis, independently induce metabolic disorders, providing experimental evidence in mice of how gut microbial imbalance fosters metabolic diseases. These findings may help inform our understanding of how the gut microbiome mediates host pathophysiology in obesity and type 2 diabetes. Consistent with this notion, serum H₂S levels were markedly elevated in the DSV, *phsABC*, and NaHS groups, accompanied by decreased expression of the colonic tight junction proteins ZO-1 and Occludin. These changes suggest a potential route through which gut-derived H₂S may gain access to the systemic circulation.

The liver, as a central organ of energy metabolism [24], appears to be one target of H₂S exposure in our mouse model. We observed significant liver weight gain in H₂S-treated mice, accompanied by a dual accumulation of glycogen and fat. The abnormal glycogen deposition in the liver and skeletal muscle, as well as the mild focal inflammatory changes in the liver, corroborated the observed phenotype of glucolipid metabolic dysregulation under H₂S exposure. These findings align with previous studies showing that liver fat accumulation and glycogen storage are key features of metabolic dysfunction in conditions [25–27]. Our transcriptomic analysis revealed that exogenous H₂S significantly suppressed major metabolic pathways in the liver: PPAR signaling, fatty acid degradation and elongation pathways, shedding light on the molecular basis for the lipid and glucose metabolic disturbances observed in our mouse model.

The PPARs are a family of nuclear receptor transcription factors that govern lipid metabolism, glucose transport, and energy homeostasis [27]. Diminished activity of these receptors impairs the proper expression of downstream metabolic gene clusters [28]. In our study, exposure to H₂S led to significant downregulation of PPAR itself and its target genes involved in fatty acid oxidation and degradation, including *Elovl2*, *Abhd2*, *Acot1*, *Acot2* [29], and *Ehhadh* [30]. These genes are essential for the breakdown and catabolism of fatty acids. Accordingly, we observed pronounced suppression of pathways related to unsaturated fatty acid biosynthesis, fatty acid elongation, fatty acid degradation, and overall lipid metabolism. This broad downregulation of lipid metabolic pathways is likely to compromise the liver’s capacity for efficient lipid clearance, thereby promoting lipid accumulation. Furthermore, when lipid metabolism is impeded, intermediates such as acylcarnitines [31] and ceramides [32] may accumulate in the cytoplasm. These lipotoxic molecules can generate negative feedback signals that further suppress PPAR expression and activity [33]. Beyond lipid accumulation, inhibition of PPAR signaling may also contribute to impaired glucose handling. PPARs are known to regulate the expression of genes involved in glucose homeostasis, and their suppression has been linked to impaired insulin signaling [34]. Thus, the downregulation of PPAR observed in our model may contribute to glucose intolerance by disrupting normal insulin sensitivity. Collectively, these findings suggest that H₂S-mediated inhibition of PPAR may represent one of the mechanisms through which H₂S disrupts lipid and glucose metabolism in our experimental model.

To further explore the functional involvement of H₂S in PPAR signaling, we examined the interplay between H₂S exposure and PPARα protein expression both in vitro and in vivo. In HepG2 cells, NaHS treatment markedly reduced PPARα protein levels and impaired cellular glucose uptake. Co-administration of the PPARα agonist OEA successfully reversed both the downregulation of PPARα and the defect in glucose uptake, indicating that H_2_S-induced metabolic dysfunction is functionally linked to impaired PPARα activity. These in vitro findings were recapitulated in mouse model: OEA intervention counteracted NaHS-induced weight gain, normalized the adipose depot weights, and restored oral glucose tolerance. Collectively, these data indicate that activation of PPARα-related signaling can mitigate several NaHS-associated metabolic abnormalities. Nevertheless, given the well-established role of H_2_S as a mitochondrial respiratory chain inhibitor, we acknowledge that PPARα suppression might also represent a secondary downstream consequence of mitochondrial dysfunction. Future studies are warranted to further delineate whether H_2_S directly targets PPARα or acts predominantly through mitochondrial pathway perturbations.

Previous studies have already revealed the pathogenic effects of H₂S from the perspectives of intestinal lipid absorption, endocrine hormone secretion, and so on. Specifically, immune surveillance restricts the outgrowth of intestinal sulfate-reducing bacteria through the T-cell–Myd88 axis. An H₂S-enriched microenvironment enhances intestinal lipid uptake via CD36 upregulation on enterocytes [14]. Concurrently, H₂S suppresses GLP-1 release from L cells by inhibiting mitochondrial respiration and triggering the IRE1α-dependent unfolded protein response, which in turn attenuates satiety signals and glucose-stimulated insulin secretion [16]. Integrating the evidence, we postulate a potential mechanistic framework for H₂S-associated metabolic alterations in mouse models, in which increased intestinal absorption, hormonal dysregulation, and impaired hepatic catabolism may act synergistically to amplify a single microbial metabolite signal into systemic positive energy balance and glucolipotoxicity.

Our study also opens up exciting avenues for therapeutic interventions aimed at alleviating metabolic disorders. The identification of H₂S-degrading probiotics is a particularly promising strategy for combating the negative effects of excessive intestinal H₂S. Probiotic species such as *Lactobacillus* [35, 36] and *Bifidobacterium* [37], which can compete with sulfate-reducing bacteria and degrade H₂S, may offer a potential avenue for further investigation in mitigating metabolic dysfunction in preclinical models. Recent studies have highlighted the capacity of these probiotics to regulate H₂S production, thereby restoring metabolic homeostasis [38]. The development of engineered probiotics capable of efficiently metabolizing or oxidizing sulfides could offer a conceptual basis for developing targeted approaches that may eventually be explored for obesity, type 2 diabetes, and other metabolic disorders if validated in future studies.

There are several limitations to this study that could guide future research. Firstly, the use of NaHS as a direct H₂S donor may not fully replicate the continuous, local production of H₂S by gut microbiota. Future studies should explore more sophisticated, sustained-release H₂S donors to more accurately simulate physiological conditions. Secondly, while we utilized engineered *E. coli* expressing the *phsABC* thiosulfate reductase complex to evaluate enzymatic H_2_S production, this system relies on thiosulfate rather than the native sulfate reduction pathway (*dsrAB*) utilized by primary sulfate-reducing bacteria like *Desulfovibrio*. Thus, *phsABC* serves as a proof-of-concept surrogate tool for local intestinal H_2_S generation rather than an exact physiological model of native SRB activity. Lastly, direct measurement of cecal and luminal H_2_S concentrations was not performed, and direct quantification of the gut-to-liver sulfide flux was not feasible in mice due to limited portal blood volume. Given the rapid turnover and high volatility of H_2_S in biological matrices, elevated serum levels may not fully reflect local intestinal dynamics or direct hepatic exposure due to mucosal metabolism and systemic clearance. Future studies employing advanced in situ microsensors or stable isotope tracing approaches are warranted to better delineate sulfide translocation along the gut-liver axis.

In conclusion, this study provides experimental evidence in mice supporting the concept that gut microbiota-derived H₂S is associated with obesity and glucose metabolism disorders. Our findings suggest that reduced hepatic PPARα expression and altered lipid metabolism-related transcription may contribute to the observed hepatic lipid accumulation and impaired glucose tolerance in our mouse model. These findings provide valuable insights into microbiome-host interactions and open new avenues for therapeutic interventions targeting the microbiome. The identification of probiotics capable of degrading H₂S offers a promising strategy for treating metabolic diseases by restoring intestinal microecology and improving metabolic homeostasis.

## Supplementary Material

Supplementary_files_wrag226

## Data Availability

The raw sequence data reported in this paper have been deposited in the Genome Sequence Archive in National Genomics Data Center, China National Center for Bioinformation / Beijing Institute of Genomics, Chinese Academy of Sciences (CRA048724) that are publicly accessible at https://ngdc.cncb.ac.cn/gsa.
