# Supplementary material for: Gut microbial H2S promotes metabolic dysfunction in mice via hepatic PPARα suppression
Source: ISME J. 2026 Sep 2;20(1):wrag226. doi: 10.1093/ismejo/wrag226 (PMC13768757; doi:10.1093/ismejo/wrag226)
Supplement: Supplementary file 1 [file supplementary_files_wrag226.zip › Supplementary_data_FigS_wrag226.docx]

**Supplementary data FigS1**. Expression of sulfur metabolism‑related genes in human cohort metagenomes. (A–J) Relative abundances of sulfur metabolism‑associated KEGG orthologs in fecal metagenomes from healthy controls (NOB) versus obese (OB) individuals: (A) K08352 *phsA*; (B) K08353 *phsB*; (C) K08354 *phsC*; (D) K11180 *dsrA*; (E) K11181 *dsrB*; (F) K02439 *glpE*; (G) K07153 *hflD*; (H) K01011 *TST*/*MPST*/*sseA*; (I) K04487 *iscS*; (J) K04085 *tusA*. (K–T) Relative abundances of the same KEGG orthologs in fecal metagenomes from individuals without (NGMD) versus with glucose metabolism disorders (GMD): (K) K08352 *phsA*; (L) K08353 *phsB*; (M) K08354 *phsC*; (N) K11180 *dsrA*; (O) K11181 *dsrB*; (P) K02439 *glpE*; (Q) K07153 *hflD*; (R) K01011 *TST*/*MPST*/*sseA*; (S) K04487 *iscS*; (T) K04085 *tusA*. ****p < 0.0001, *p < 0.05, ns, not significant.

**Supplementary data FigS2**. Construction of animal models with H_2_S accumulation and biochemical detection (A) Experimental workflow for heterologous expression of *Desulfovibrio desulfuricans*‑derived *dsrAB* in *E. coli BL21*. (B) Experimental workflow for heterologous expression of *Salmonella typhimurium*‑derived *phsABC* in *E. coli BL21*. (C) Agarose gel electrophoresis of linearized pETDuet vector. (D) Agarose gel electrophoresis of the *dsrAB* gene amplicon. (E) Agarose gel electrophoresis of the *phsABC* gene amplicon. (F) Relative abundance of *Desulfovibrio desulfuricans* in mouse feces from control versus *D. desulfuricans*‑gavaged groups. (G) Relative abundance of the *phsABC* gene in mouse feces from control versus engineered H₂S‑producing *E. coli*‑gavaged groups. (H) Serum IL‑6 concentrations in control versus engineered H₂S‑producing *E. coli*‑treated mice. (I) Serum TNF‑α concentrations in control versus engineered H₂S‑producing *E. coli*‑treated mice. (J) Serum H₂S concentrations in control versus *D. desulfuricans*‑treated mice. (K) Serum H₂S concentrations in control versus engineered H₂S‑producing *E. coli*‑treated mice. (L) Serum H₂S concentrations in control versus NaHS‑treated mice. (M) Serum H₂S concentrations in control, NaHS, and C₇H₅BiO₄ rescue groups. (N) Univariate correlation analysis between mouse body weight and the weights of EAT, IAT, PRAT, and BAT. *p < 0.05, **p < 0.01, ***p < 0.001, ****p < 0.0001.

**Supplementary data FigS3**. Histopathological assessment of liver and intestinal barrier integrity in H₂S‑exposed mice. (A) Representative H&E staining of liver sections from control mice. (B) Representative H&E staining of liver sections from *phsABC*‑engineered *E. coli*‑treated mice. (C) Representative H&E staining of liver sections from NaHS‑treated mice. (D) Representative immunofluorescence images of mouse colonic sections from control group, showing ZO‑1 (red), occludin (green), and DAPI‑stained nuclei (blue). (E) Representative immunofluorescence images of mouse colonic sections from *phsABC*‑engineered *E. coli*‑treated group, showing ZO‑1 (red), occludin (green), and DAPI‑stained nuclei (blue). (F) Representative immunofluorescence images of mouse colonic sections from NaHS‑treated group, showing ZO‑1 (red), occludin (green), and DAPI‑stained nuclei (blue). (G) Quantitative analysis of ZO‑1 immunofluorescence intensity in mouse colonic sections from control, *phsABC*‑engineered E. coli‑treated, and NaHS‑treated groups. (H) Quantitative analysis of occludin immunofluorescence intensity in mouse colonic sections from control, *phsABC*‑engineered E. coli‑treated, and NaHS‑treated groups. ****p < 0.0001.

**Supplementary data FigS4**. H_2_S modulates hepatic lipid metabolism pathways and PPARα expression. (A) GSEA of lipid metabolism‑related gene sets, including unsaturated fatty acid biosynthesis, fatty acid elongation, fatty acid degradation, and fatty acid metabolism, in liver transcriptomes from NaHS‑treated versus control mice. (B) Representative Western blot of PPARα protein in HepG2 cells treated with NaHS (1 mM) with or without the PPARα agonist INN (100 µM). (C) Densitometric quantification of PPARα protein expression in HepG2 cells after NaHS and/or INN treatment. (D) Representative Western blot bands of PPARα protein in mouse liver tissues from control and OEA‑treated groups. (E) Representative Western blot bands of PPARα protein in mouse liver tissues from control and C₇H₅BiO₄ rescue groups. (F) Densitometric quantification of PPARα protein expression in mouse liver tissues from control and OEA groups. (G) Densitometric quantification of PPARα protein expression in mouse liver tissues from control and bismuth subsalicylate rescue groups. (H) Representative PAS staining of liver sections from control and bismuth subsalicylate rescue groups, with quantification of positive staining area and average optical density. *p < 0.05, **p < 0.01, ***p < 0.001.
